# Sonographic scoring of solid thyroid nodules: effects of nodule size and suspicious cervical lymph node^☆^

**DOI:** 10.1016/j.bjorl.2016.01.013

**Published:** 2016-04-19

**Authors:** Ozlem Unsal, Meltem Akpinar, Bilge Turk, Irmak Ucak, Alper Ozel, Semra Kayaoglu, Berna Uslu Coskun

**Affiliations:** a Sisli Etfal Teaching and Research Hospital, Head and Neck Surgery, Clinic of Otolaryngology, Istanbul, Turkey; b Sisli Etfal Teaching and Research Hospital, Clinic of Radiology, Istanbul, Turkey; c Nisantasi Family Health Center, Family Medicine, Istanbul, Turkey

**Keywords:** Thyroid malignancy, Thyroid nodule, Ultrasound characteristics, Scoring, Suspicious, Malignidade de tireoide, Nódulo tireoidiano, Características ultrassonográficas, Escore, Suspeito

## Abstract

**Introduction:**

Ultrasound is the most frequently used imaging method to evaluate thyroid nodules. Sonographic characteristics of thyroid nodules which are concerning for malignancy are important to define the need for fine needle aspiration biopsy or open surgery.

**Objective:**

To evaluate malignancy risk of solid thyroid nodules through sonographic scoring. The effects of nodule size ≥2 cm and associated pathologic cervical lymph node in scoring were examined in addition to generally excepted suspicious features.

**Methods:**

Medical data of 123 patients underwent thyroid surgery were reviewed, and 89 patients (58 females, 31 males) were included in the study. The presence and absence of each suspicious sonographic feature of thyroid nodules were scored as 1 and 0, respectively. Total ultrasound score was obtained by adding the positive ultrasound findings. Differently from the literature, nodule size ≥2 cm and associated pathologic cervical node were added in scoring criteria. The diagnostic performance of nodule characteristics for malignancy and the effect of total US score to discriminate malignant and benign disease were calculated.

**Results:**

A significant relationship was found between malignancy and hypoechogenity, border irregularity, intranodular vascularity, and microcalcification (*p* < 0.05). Pathologic cervical node was observed predominantly in association with malignant nodules. Positive predictive value of suspicious cervical node for malignancy was 67%, similar to microcalcification. Nodule size ≥2 cm was not distinctive for diagnosis of malignancy. The number of suspicious sonographic features obtained with receiver operating characteristic analysis to discriminate between malignant and benign disease was three.

**Conclusion:**

Sonographic scoring of thyroid nodules is an effective method for predicting malignancy. The authors suggest including associated pathologic node in the scoring criteria. Further studies with larger cohorts will provide more evidence about its importance in sonographic scoring.

## Resumo

**Introdução:**

A ultrassonografia é o método imagiológico mais frequentemente utilizado na avaliação de nódulos tireoidianos. As características ultrassonográficas dos nódulos tireoidianos que dizem respeito à malignidade são importantes para a definição da necessidade de uma biópsia por aspiração com agulha fina ou uma cirurgia aberta.

**Objetivo:**

Avaliar o risco de malignidade de nódulos tireoidianos sólidos por meio de escore ultra-sonográfico, verificando os efeitos de nódulos ≥ 2 cm, em associação com linfonodo cervical patológico, além de características suspeitas geralmente omitidas.

**Método:**

Foram revisados dados médicos de 123 pacientes tratados com cirurgia da tireoide. Foram incluídos no estudo 89 pacientes (58 mulheres, 31 homens). Presença e ausência de cada característica ultrassonográfica suspeita de nódulo tireoidiano receberam pontuações de 1 e 0, respectivamente. O escore ultrassonográfico total foi obtido pela soma dos achados ultrassonográficos positivos. Diferentemente da literatura, nódulos ≥ 2 cm e nodo cervical patológico associado foram acrescentados nos critérios de pontuação. Foram calculados o valor diagnóstico das características dos nódulos para malignidade e o efeito do escore ultrassonográfico total na diferenciação entre doença maligna *vs.* benigna.

**Resultados:**

Foi encontrada uma associação significante entre malignidade e hipoecogenicidade, irregularidade das margens, vascularidade intranodular e microcalcificação (*p* < 0,05). Nodo cervical patológico foi observado predominantemente em associação com nódulos malignos. O valor preditivo positivo de nodo cervical suspeito para malignidade foi de 67%, similar ao achado para microcalcificação. Diâmetro de nódulo ≥2 cm não foi fator diferenciador para diagnóstico de malignidade. O número de características ultrassonográficas suspeitas obtido com a análise da curva de características de operação do receptor (*receiver operating characteristic*, ROC) para discriminação entre doença maligna *vs.* benigna foi igual a 3.

**Conclusão:**

O escore ultra-sonográfico dos nódulos tireoidianos é método efetivo para predição de malignidade. Sugerimos a inclusão de nódulo patológico associado aos critérios de pontuação. Futuros estudos com coortes maiores proporcionarão mais evidências sobre sua importância no escore ultrassonográfico.

## Introduction

Thyroid nodules are commonly seen with malignancy rate of 10–15%.^1^ Although fine needle aspiration biopsy (FNAB) still is the most reliable test for malignancy detection with >95% of accuracy, the indeterminate cytology was observed in 15–30% of FNABs.^1^, ^2^ Most lesions showing indeterminate cytology happened to be benign after histopathological evaluation of surgical specimens.^3^

Ultrasonography (US) was proven to be a reliable and easily available diagnostic method with high sensitivity (90%) and specificity (85%) for thyroid nodules.^4^ The sonographic characteristics of thyroid nodules that suggest malignancy include hypoechogenicity, solid structure, irregular margin, microcalcification, and regional lymph node metastasis.^5^, ^6^, ^7^, ^8^ Intranodular hypervascularity and nodule size ≥2 cm were also considered as indicators of thyroid malignancy in the literature.^9^, ^10^, ^11^

US-based classification methods have been used by several researchers to evaluate thyroid nodules.^12^ However, the roles of suspicious cervical lymph node (LN) and nodule size ≥2 cm in sonographic scoring of thyroid nodules had not been analyzed in a same study together. In this study, the authors assessed the feasibility and efficacy of these features on sonographic scoring to distinguish malignant and benign thyroid nodules.

## Methods

### Patients

The records of 123 patients (58 females, 31 males) who underwent thyroid surgery (lobectomy, isthmolobectomy, or total thyroidectomy) between February 2013 and July 2014 in Otolaryngology Clinic at Sisli Etfal Teaching and Research Hospital were reviewed. Five patients with hyperthyroidism, four patients with incomplete US images, five patients with inconclusive cytopathology result, 11 patients with revision surgery, and nine patients with pure cystic nodule were excluded from the study. The age, gender, pre-operative US reports, FNAB results and histopathology results of surgical specimens of 89 patients were recorded. The institutional research ethics committee approved the present study under No. 783, and an informed consent was obtained from all patients.

### Ultrasound

Gray scale ultrasound and color Doppler study were performed by radiologists experienced on neck US with a high-resolution ultrasound (Siemens S2000 – Erlangen, Germany) equipped with a 5–14 MHz linear probe.

Only solid nodules were included in the study. A solid thyroid nodule was described as merely solid or predominantly solid when 10% or less of the total volume was cystic. Suspicious sonographic nodule characteristics including marked hypoechogenicity, irregular margins, microcalcifications, intranodal vascularity, associated cervical LN with intranodal cystic components, or microcalcifications and nodule size ≥2 cm in the longest diameter were evaluated. In the presence of multiple thyroid nodules, the nodule with the largest size and/or with the highest malignancy potential at sonographic findings was selected.

Marked hypoechogenicity was described as having the same or decreased echogenicity regarding strap muscles. A nodule margin including microlobulation and/or a spiculated border may be related to thyroid malignancy. Microlobulation was described as the presence of small lobules on the surface of a nodule; a spiculated border was described as the presence of irregular spiculation located on the surface of a nodule. Microcalcifications were observed as small, hyperechoic foci (<1 mm in size), presenting no comet-tail artifacts or posterior shadow. Intranodal vascularity determined with color Doppler US was described as flow in the central part or in both central and peripheral part of the nodule.

### Scoring of suspicious characteristics

In US images and reports, the presence of each suspicious feature of evaluated nodule-hypoechogenity, microcalcification, border irregularity, size ≥2 cm, intranodal vascularity, and associated pathologic cervical LN, were scored as 1, while the absence was scored as 0. The total US score was obtained by adding each individual score of suspicious sonographic features. The total US score was separated into two groups (low and high) based on the optimal cut-off value calculated using receiver operating characteristic (ROC) analysis.

#### Fine needle aspiration biopsy

US-guided FNABs were performed by experienced radiologists using a 27-gauge needle attached to a 10-mL disposable plastic syringe and aspirator. No local anesthesia was applied. Each nodule was carefully aspirated at least twice. After FNAB, the collected specimen was smeared on glass slides and fixed with 95% alcohol for Papanicolaou staining.

#### Histopathological evaluation

All thyroidectomy specimens were made available for histopathological examination. Tissue samples were immersed into paraffin blocks, from which 3–5 μm ultrathin sections were obtained. The sections were stained with hematoxylin and eosin. Special stains were performed when required. All smears and sections were examined by experienced pathologists. The final diagnosis of the nodules was reached through the specimen histopathology.

### Statistical analysis

Statistical analysis was performed with SPSS 15.0 software package for Windows (SPSS, Inc., Chicago, IL). Descriptive statistics were given as numeric and percentage for categorical variables. The dependent ratio of categorical variables among the groups was tested using MC Nemar analysis whereas independent ratio was analyzed with chi-squared test. When conditions of comparisons could not provide the proportions of independent groups, a Monte Carlo simulation was applied. The Kappa coefficient was used as a measure of concordance in the applied protocol, considering: <0, no concordance; 0.0–0.20, low concordance; 0.21–0.40, moderate concordance; 0.41–0.60, high concordance, 0.81–1.00; very high concordance. The statistical significance was defined as *p* < 0.05.

The optimal cut-off value for the total US score to differentiate malignant and benign solid nodules was obtained with ROC analysis at the maximum sensitivity and specificity.

## Results

Thirty-one male (34.8%) and 58 female (65.2%) patients aged between 21 and 82 (median 51.9 ± 13.1) were included in the study. The frequency of each suspicious feature and total US scores of nodules are summarized in Table 1. Size ≥2 cm, hypoechogenity, and microcalcification were the most commonly encountered suspicious sonographic features among all nodules, with incidence rates of 68.5%, 33.7%, and 20.2%, respectively.

**Table 1:** Suspicious sonographic features, total ultrasound (US) scores, and histopathology results of evaluated thyroid nodules with incidence rates.

	*n*	%
*Hypoechogenity*	30	33.7
*Size ≥2* *cm*	61	68.5
*Border irregularity*	11	12.4
*Intranodal vascularity*	16	18.0
*Microcalcification*	18	20.2
*Suspicious lymph node*	3	3.4

*Total US score*		
0	7	7.9
1	45	50.6
2	20	22.5
3	12	13.5
4	5	5.6

*High US score*		
≥3	17	19.1

*Low US score*		
<3	72	80.9

*Histopathology*		
Malignant	29	32.6
Benign	60	67.4

The cut-off value of total US score to discriminate malignant and benign nodules was 3 (Az = 0.675) based on ROC analysis at maximum sensitivity and specificity. The total US score of nodules were separated into two groups as low (<3) and high (≥3) US scores according to that cut-off value. The incidence rates of low and high scored nodules and the histopathological results of specimens can also be observed in Table 1. Seventy-two of 89 nodules (80.9%) scored under 3, whereas 17 (19.1%) nodules had high US score (≥3). The final histopathology results were malignant in 29 (32.6%) and benign in 60 (67.4%) of the nodules after surgical removal. Histopathologic examination of malignant nodules revealed 28 (96.6%) papillary carcinomas and one (3.4%) lymphoma.

The malignancy rates in high and low scored nodules are presented in Table 2. Seventeen nodules were located in the high score group, and 15 (82.2%) of them revealed malignancy, whereas 14 nodules out of 72 (19.4%) in the low score group presented malignancy. The malignancy risk of high scored nodules was significantly higher compared to nodules with low scores (*p* = 0.004).

**Table 2:** Distribution of malignant and benign nodules in the low and high score groups.

	Histopathology of nodules					
	Malignant		Benign		*p*	Kappa
	*n*	%	*n*	%		
*Total US score*					0.004	0.542
High (≥3)	15	88.2	2	11.8		
		51.7		3.3		
Low (<3)	14	19.4	58	80.6		
		48.3		96.7		
	29		60			

When suspicious nodule characteristics including hypoechogenity, border irregularity, intranodular vascularity, and microcalcification were compared with histopathology results, the malignancy risk was found to be significantly higher in the nodules with these sonographic features, when compared with those without them (*p* < 0.05; Table 3).

**Table 3:** Malignancy rates of the nodules with and without suspicious sonographic features.

	Histopathology of nodules				*p*
	Malignant		Benign		
	*n*	%	*n*	%	
*Hypoechogenity*					0.003
+	16	53.3	14	46.7	
−	13	22.0	46	78.0	

*Border irregularity*					0.019
+	7	63.6	4	36.4	
−	22	28.2	56	71.8	

*Hypervascularity*					0.026
+	9	56.3	7	43.8	
−	20	27.4	53	72.6	

*Microcalcification*					0.001
+	12	66.7	6	33.3	
−	17	23.9	54	76.1	

Nodule size ≥2 cm was not distinctive for the diagnosis of malignancy in this study (*p* > 0.05). Contrarily, it was detected that the nodules ≥2 cm were associated with benign histopathology. However, the distribution of the nodules sized ≥2 cm among high and low scored nodules was similar. No significant differences were demonstrated (*p* > 0.05)

Three cervical LNs with suspicious features were reported sonographically in the present study, and two of them (66.6%) were associated with thyroid malignancy.

For each suspicious sonographic feature, the calculated diagnostic performances including sensitivity, specificity, positive predictive value (PPV), negative predictive value (NPV) and accuracy, which are depicted in Table 4. Microcalcification and presence of suspicious LN had the highest PPV (0.667).

**Table 4:** Diagnostic performances of suspicious sonographic characteristics of thyroid nodules.

	Sensitivity (%)	Specificity (%)	PPV (%)	NPV (%)	Accuracy
Ultrasound	0.517	0.967	0.882	0.806	0.802
Hypoechogenity	0.552	0.767	0.533	0.780	0.764
Size ≥2 cm	0.483	0.217	0.230	0.464	0.303
Border irregularity	0.241	0.933	0.636	0.718	0.708
Hypervascularity	0.310	0.883	0.563	0.726	0.697
Microcalcification	0.414	0.900	0.667	0.761	0.742
Suspicious lymph node	0.069	0.983	0.667	0.686	0.685

## Discussion

In adults, the prevalence of thyroid nodules was reported as 50%.^13^ Ultrasonographic examination, which is the main diagnostic modality for the detection of thyroid nodules, increased the identification rate of clinically asymptomatic thyroid nodules, with a prevalence of 19–67%.^14^ However, its ability to differentiate between malignant and benign nodules is still unclear and a consensus has not been reached yet.

US-guided FNAB is the diagnostic method for the histopathological evaluation of thyroid nodules. However, there are some disadvantages, such as inadequate sampling, indeterminate cytology, cost, invasiveness, and operator dependency.^12^ At this point, the use of ultrasonographic features to predict malignancy may decrease the need for FNAB, but to date, none of the sonographic nodullary features was found to be 100% sensitive or specific for thyroid malignancy.

The sonographic suspicious features considered in this study to differentiate the potential malignant solid nodules were based on some previous studies.^12^, ^14^, ^15^ The presence of hypoechogenity, microcalcification, border irregularity, and intranodal hypervascularity was assessed. Differently from the literature, the presence of suspicious cervical LN and nodule size ≥2 cm were included in the scoring criteria in this study.

Prelaryngeal, pretracheal, and paratracheal LNs are commonly observed sites of thyroid carcinoma metastasis. Cervical LN features, including hyperechoic punctuations, cystic appearance, peripheral vascularization, and loss of hilum, have been associated with malignancy.^16^ A thyroid nodule co-existing with a cervical LN presenting the aforementioned characteristics is a remarkable sonographic finding regarding malignancy. In this study, suspicious cervical LN was encountered in three patients. Two of them accompanied thyroid carcinoma. A PPV of 66.7% for cancer was observed in the presence of suspicious cervical LN, similarly in microcalcified nodules. Therefore, the authors believe that the inclusion of suspicious LN in the scoring criteria has an important role in predicting thyroid malignancy. However, further studies are required due to the inadequate number of suspicious cervical LNs to understand its importance in terms of sonographic scoring and predicting thyroid malignancy.

Although nodule size is considered a non-specific finding, a previous study revealed that there was a non-linear relationship between size and malignancy rates. The approximate threshold for increased malignancy was found to be 2 cm.^10^ In addition, Smith-Bindman et al.^11^ reported that three US characteristics – nodule size ≥2 cm, microcalcification and solid composition – were closely associated with thyroid carcinoma risk. Therefore, the authors decided to include the nodule size ≥2 cm in scoring criteria. However, it was observed that the distribution of nodules ≥2 cm in both high and low score groups were similar, and that this distribution was not statistically significant (*p* > 0.05). Thus, nodule size ≥2 cm was considered not to have an important role in scoring. Although nodule size ≥2 cm was found to be statistically insignificant, in practice there is a tendency to sample from the largest nodules in the thyroid gland. Moreover, the revised 2009 American Thyroid Association Guidelines suggests to sample from the largest nodule in the presence of two or more nodules if none has suspicious sonographic appearance and the nodules are sonographically similar.^1^

Predictors of thyroid malignancy including microcalcification, marked hypoechogenicity, and irregular margins have been also reported.^15^, ^16^ Microcalcification, despite of low sensitivity, is a relatively specific ultrasonographic indicator of thyroid carcinoma. In a previous study, a total of 45–60% of malignant nodules showed microcalcifications *versus* 7–14% of benign nodules.^16^ Ahn et al.^17^ reported a PPV pf 85.1% for cancer with the presence of microcalcifications. In another study, 60% of patients with microcalcifications were found to have malignant disease.^18^ The present study observed a PPV of 66.7% for cancer in microcalcified nodules, the highest PPV, similarly in the presence of suspicious cervical LN.

Malignant nodules are frequently seen to be markedly hypoechoic.^16^ Cappelli et al.^19^ found a 3.8 odds ratio of malignancy in solid hypoechoic nodules in a prospective study of 349 surgically excised thyroid nodules. The present study found a 2.8 odds ratio of malignancy in hypoechoic nodules.

Border irregularity of a thyroid nodule is considered to be notable sonographic feature. The increased malignancy risk related to irregular margins has been debated in the literature.^16^ In the present study, irregular border was detected in 11 of 89 nodules. Seven of them (7/11; 63.6%) were found to be malignant, whereas four nodules (4/11; 36.4%) were benign. Border irregularity was found to be statistically higher in malignant nodules than in benign (*p* = 0.019).

Nodule vascularity is generally evaluated with the use of color Doppler US. It has been demonstrated that 42–74% of malignant thyroid nodules present hypervascularity and a prominent central blood flow.^20^ Frates et al.^21^ reported that a centrally predominant vascularity was encountered in malignant nodules (42%) more often than in benign nodules (14%). Similarly, the present study revealed a hypervascularity rate significantly higher in malignant nodules when compared with benign nodules (*p* = 0.026).

Various reporting and data systems based on sonographic features have been discussed to identify malignancy risk. Horvath et al.,^22^ with a modified recommendation from Jin Kwak et al.,^23^ proposed the Thyroid Image Reporting and Data System (TIRADS) in order to improve patient management and cost-effectiveness by avoiding unnecessary FNAB of thyroid nodules. However, its clinical use is very limited.

According to the guidelines of the American Association of Clinical Endocrinologists, the co-existence of at least two suspicious US features increases thyroid cancer risk.^24^ Ozel et al.^25^ suggested that at least three US features are considered for malignancy risk in nodules sized <1 cm with diagnostic accuracy of 89.9%, whereas two US features are considered for nodules sized ≥1 cm with the diagnostic accuracy of 93.8%. In the present study, the cut-off value for suspicious sonographic features including size ≥2 cm and presence of suspicious cervical LN was three (Az = 0.675), and the diagnostic accuracy of US was 80.2%.

The sonographic scoring of thyroid nodules may increase diagnostic accuracy of US for thyroid malignancy and prevent unnecessary invasive tissue sampling and open surgical procedures, and also provides a quick method to decide which nodule to be sampled.

There are some limitations in this study. The first is that only one suspicious thyroid nodule for each patient was selected. The second is that the retrospective images might be limited, providing inadequate information compared to a dynamic study where more extended analysis can be performed. Furthermore, in the present series, the high incidence of papillary thyroid carcinoma (96.5%) may lead to a bias, since generally accepted suspicious US features are not enough to detect follicular carcinoma.

## Conclusion

Ultrasound is a non-invasive and effective tool for evaluation of thyroid nodules. The scoring of suspicious features based on US is practical to decide which nodule needs to be sampled. Contrary to nodule size, the authors believe that the presence of suspicious cervical LN should be included in generally accepted scoring criteria in an attempt to predict thyroid malignancy even though an adequate number of suspicious LN for an accurate statistical analysis could not be achieved in this study. Larger cohorts may provide more prominence to the association between suspicious cervical LN and thyroid malignancy risk.

Although none of the suspicious sonographic characteristic offers sufficient specificity and sensitivity to diagnose malignancy, their cumulative effect highlights a potential thyroid malignancy. In this study, three or more suspicious sonographic features indicated a potential malignant thyroid nodule. However, further prospective cohort studies based on real time US are needed for more accurate results.

## Conflicts of interest

The authors declare no conflicts of interest.
